# Osteopontin (SPP1) expression on gallstone formation in rabbits fed with a lithogenic diet

**DOI:** 10.5455/javar.2023.j682

**Published:** 2023-06-30

**Authors:** Tareek Abdulqadir Abdal, Raed Salim Al-Naemi

**Affiliations:** 1 Department of Theriogenology, Physiology, and Anatomy, College of Veterinary Medicine, University of Duhok, Duhok, Iraq; 2 Department of Physiology, College of Medicine, University of Duhok, Duhok, Iraq

**Keywords:** Osteopontin, gene expression, gallbladder stone, cholelithiasis, oxidative stress, lithogenic diet

## Abstract

**Objective:**

This research was designed to assess the influence of the administration of a lithogenic diet, hydrogen peroxide, and vitamin AD3E on rabbits’ gallstone formation and to envisage the expression of osteopontin (OPN) in their hepatic tissues.

**Materials and Methods:**

Twenty-four healthy local mature rabbits of both genders were divided into four equal groups. At the end of the feeding period, samples of blood were taken from all rabbits after they had fasted overnight to estimate the serum lipid profile. And some of the hepatic tissue has been preserved at −28°C for molecular analysis and gene expression.

**Results:**

The gallstones were formed 100% in the GIII and 50% in the GIV. The mRNA OPN expression showed a significant increase in the GIII when compared with other groups. In Groups III and II, the serum levels of total cholesterol, Triglyceride, L-C, low-density lipoprotein-choles, and VLDL-C were significantly increased when compared with GI, while in GIII, the serum level of high-density lipoprotein-cholesterol was significantly decreased when compared with GI.

**Conclusion:**

It was concluded that the expression of the mRNA OPN was increased in the hepatic tissue of gallstone-formed rabbits.

## Introduction

In 1979, Osteopontin (OPN) was first noticed by Senger, and in 1985 was named by Franzen [1], OPN is a Latin word osteo means bone and pontin means Bridge, which has a relation with the lusty role of OPN in the body, sometimes OPN mentioned as an early T-lymphocyte activation protein 1 [2], or secreted phosphoprotein 1 (SPP-1) [2,3], OPN is encoded by SPP1 gene that is a multifunctional soluble cytokine and a matrix-related glycoprotein belongs to the small integrin-binding ligand N-linked glycoprotein [4,5], existing in some fluids of the body such as blood, urine, and milk, and a plurality of tissues [6] and in many organs like gall bladder, kidney, lungs, breasts, brain, placenta, salivary and sweat glands, pancreas, inner ear, arteries, nerve cell, an immune cell, urinary and reproductive tracts, epithelial cells of the digestive system [7,8], OPN has participated in a different number of physiological processes including the organizing of bone formation/resorption, organizing of the immune system, brain and gut development [6], OPN has also a pathogenic role in atherosclerosis, tumorigenesis, metastasis, and liver inflammation and injury [7,1], it canchange the hepatic cholesterol metabolism thus participating in the pathogenesis of cholelithiasis in experimental animals [9], and because of its increased expression in the epithelium of intrahepatic bile stone-loaded ducts, it is closely related to hepatolithiasis [10]. However, the mechanism and role of OPN in forming cholesterol gallstone (GS) are undetermined [11].

Gallstone disease (GDs) is considered a great health issue worldwide because of its related complications and comorbidities. It has a large financial burden on the healthcare economy, and GDs are influenced by an interaction of complicated environmental and genetic factors [12]. About 10%–15% of the global adult population is influenced by GDs because it is a highly prevalent gastroenterological disorder [13,14]. It is not lethal, but it can result in severe GIT disorders leading to many serious complications and outcomes, such as pancreatitis, gallbladder cancer, and acute GSs [15]. The pathogenesis and formation of cholesterol gallstone disease (CGD) have long been mysteries [16,17]. It has been thought for many years that GD was due to an inflammatory disease of the gallbladder tissues (cholecystitis), which leads the cell to desquamate and produce abnormal materials. For the time being, the hepatic production of cholesterol-hypersaturated bile is considered a precondition and prerequisite for CGD [17,18]. Depending on the major chemical ingredients, GSs are classified into three kinds: cholesterol GSs, pigmented GSs, and mixed GSs, and anatomically, GSs can be categorized as intrahepatic and extrahepatic bile duct stones [19]. An imbalance between major bile ingredients, cholesterol, phospholipids, and bile salts, is considered the main factor resulting in GS formation [20]. The supersaturation of bile and the precipitation of cholesterol are required when there is a shortage of salts or phospholipids and too much cholesterol or when there is a combination of these factors so that the homeostasis of cholesterol is important to stop the formation of GSs. Cholesterol homeostasis is maintained when there is an equilibrium between intake, biosynthesis, and biliary secretion of cholesterol [21].

Oxidative stress is determined as an unstable equilibrium between the free radical generation and the antioxidant capacity of the cells [22], causing potential cellular prejudice toward proteins, fat, nucleic acids, and carbohydrates [23]. It has been studied since the 1970s, but its concept can be traced back to the 1950s [24]. Free radicals are usually known as any chemical species that exist independently and are usually unstable and highly reactive chemical species due to the presence of one or more unpaired electrons in the outer orbitals; therefore, they are characterized by high reactivity in chemical reactions for electron exchange [25]. There are two sorts of free radicals in the biological system, reactive oxygen species (ROS) and reactive nitrogen species [26,27], that are produced in the liver as a byproduct through the processes of metabolizing various compounds [28,29] and also through a mitochondrial electron chain reaction [30]. Hydrogen peroxide (H_2_O_2_) is capable of causing significant cellular damage even at low concentrations [31]. Under normal physiological conditions, the body has enough antioxidant defense capacity and repair systems [32–34] to recognize and remove molecules damaged by free radical oxidation. A mild degree of oxidative stress can be resisted by most body cells [35]. The equilibrium between the elimination rate of free radicals by specific antioxidants and their generation rate is important [36], because impairment or limitation in the action of antioxidants and the excessive amounts of cellular free radicals production resulting in the excessive ROS generation can be harmful, which depletes the endogenous antioxidants that subsequently fail to counteract all the ROS, exerting deleterious effects on cell function because they are highly reactive with all biological substances such as proteins, nucleic acids, lipids, damaging proteins, DNA, and lipid peroxidation [37,38], and degradation of poly-unsaturated fatty acids occurs by ROS forming malondialdehyde, which is considered a reliable and popular biomarker to estimate and measure oxidative stress levels in an organism [39–41].

## Materials and Methods

### Ethical approval

In this research, all experimental works were approved by the Local Ethics Committee set up at the College of Veterinary Medicine, University of Duhok (No. CVM20190910UD).

### Chemicals

Dihydrocholesterol (Alfa Aesar Thermo Fisher, Scientific, Germany); H_2_O_2_ (Scharlab S.L., Gato Perez, Sentmenat, Spain). Vitamin AD3E injection (interchemie, Netherlands) total Ribonucleic acid (RNA) extraction kit supplied by ADD BIO-KOREA, complementary deoxyribonucleic acid (cDNA) supplied by ADD BIO-KOREA, SPP1 primer, and Glyceraldehyde-3-phosphate dehydrogenase (GAPDH) primer (Macrogene/Korea).

### Animals, diets, and treatment

Twenty-four healthy local mature rabbits, regardless of sex, were used in this study, with weights between 1 and 1.5 kg. All rabbits were environmentally adapted in the animal holding room and kept under standard laboratory conditions for at least a week prior to the experiment; they were put in cages and fed with standard locally prepared diets with free access to tap water. Regarding their sex, they were divided into four equal groups, consisting of three males and three females separated from each other, and every individual rabbit was housed in a stainless steel cage. G1 (control group): rabbits in this group did not receive any medications; they were fed a standard rabbit diet and water for 6 weeks. GII (H_2_O_2_ group): in this group, rabbits were administered 1% H_2_O_2 _with their drinking water, which was prepared daily in a dark bottle, and a normal diet for 6 weeks. GIII (lithogenic group), in addition to 1% H_2_O_2 _in their drinking water, was fed a lithogenic diet (LD) consisting of 1% dihydrocholesterol, which begins at the start of the 4th week and continues until the end of the 6th week for the induction of GSs. In the GIV (Vit. AD3E group), in this group, the same treatments were as in GIII except for stopping 1% H_2_O_2 _administration. The rabbits were also injected with 0.1 ml of vitamin AD3E twice weekly from the start of the 4th week and continued till the end of the 6th week to prevent or reduce gallstone occurrences.

### A sampling of the blood and hepatic tissues

**Figure 1.**
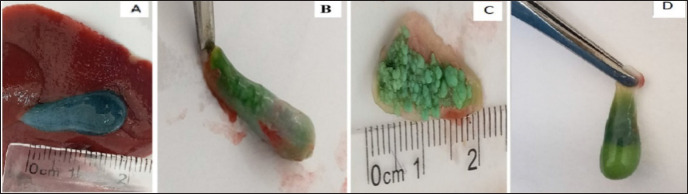
Induced gallbladder stone formation experimentally in rabbits. A = normal gallbladder in the GI. B and C show the induction of GSs in GIII. D shows partial recovery from stone formation in GIV.

**Figure 2.**
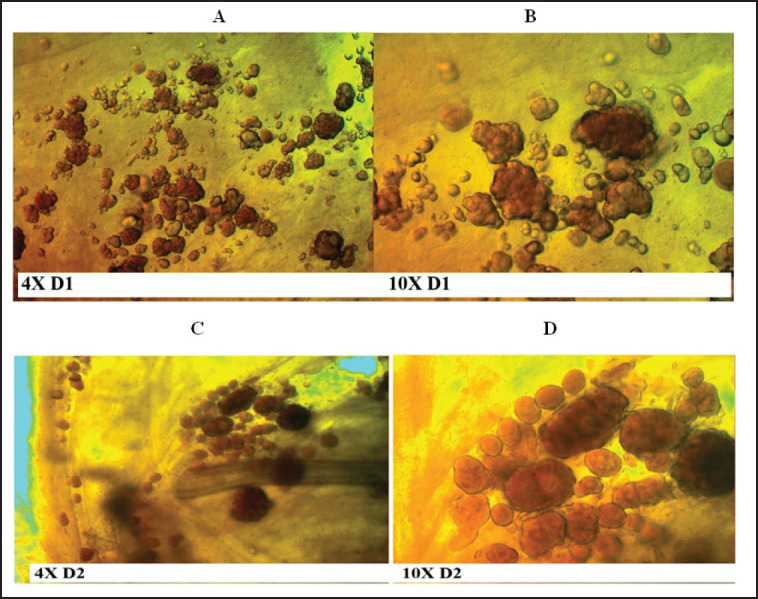
Microscopic appearance under two magnifications power (4 and 10×) of the opened gallbladder cyst filled with stones of two rabbits in GIII (number D1 and D2). *D = Dihydrocholesterol.

According to the ethical guidelines that were approved by the American Veterinary Medical Association, all animals are euthanized before killing. After an overnight fast, blood was immediately and directly drawn from the heart into a heparinized test tube at the end of the feeding period (6 weeks). By centrifuging the blood at 3,500 rpm for 20 min, the isolation of serum was done. The separated serum samples were immediately put in an Eppendorf tube and stored at −20°C for measurement of biochemical parameters. All rabbits were sacrificed, and an immediate autopsy was performed for each rabbit in order to obtain the stone, hepatic, and gallbladder samples. A cholecystectomy was performed, and each gallbladder was cleaned trimmed of extraneous tissue, and washed with normal saline, and the bile was withdrawn by a sterile disposable syringe in all rabbits in the GIII. It is difficult to withdraw their bile because their gallbladder is filled with stones (Fig. 1B). After the bile has been drawn, each gallbladder has been opened to notice the GSs. A piece of the gallbladder has been put on a slide to take an immediate image showing the gallbladder tissues and the presence of any remaining GSs under a different magnification lens (Fig. 2A–D) and some of the hepatic tissue has been preserved at −28°C for molecular analysis and gene expression.

### Serum lipid profile

A standard commercial kit provided by Roche (USA): Serum total cholesterol (TC): REF: 03039773; serum Triglyceride (TG): REF: 20767107 322. Serum low-density lipoprotein-cholesterol (REF: 03038866) and serum high-density lipoprotein-cholesterol (REF: 07528566) were used to estimate the lipid profile.

### Quantitative real-time reverse transcription-polymerized chain reaction (PCR) analysis

According to the instructions of the company, the total RNA from 50 mg of frozen hepatic tissue was extracted using the total RNA extraction kit supplied by ADD BIO-KOREA. 2 μl of total RNA was used to generate first-strand complementary DNA (cDNA) with ADD BIO-KOREA. Subsequently, 5 μl of cDNA product was used for PCR to amplify OPN with the corresponding primers, SPP1: 5′-TCT CCT AAC ACC GCA GAA TG -3′ (Forward) and 5′-TCT GTA AGC CAC ACT GTC AC-3′ (Reverse), GAPDH: 5′-GGC GTG AAC CAC GAG AAG TA-3′ (Forward) and 5′-TCC ACA ATG CCG AAG TGG TC-3′ (Reverse) [42]. The Amplicon sizes of PCR products were 310 and 116 bp, respectively. The temperature cycling protocol consisted of 1 cycle of a preheating step (95°C for 5 min), 1 cycle of denaturation (94°C for 45 sec), 30 cycles of annealing (55°C for 45 sec), and 1 cycle of extension (72°C for 45 sec). A final extension step (72°C for 5 min) was also carried out. Experiments were performed in duplicate, and normalized mRNA expression was done by GAPDH. In a 1% agarose gel, the separation of the PCR products occurred. The band intensity was quantified by ImageJ Software. Using ImageJ Software, the intensification of the SPP1 gene band was normalized with that of the reference gene GAPDH.

### Statistical analysis

All statistical analyses were performed using one-way analysis of variance, and differences between groups were estimated using Duncan multiple range tests using version 23 of SPSS. The results were expressed as the mean ± standard error of the mean of six rabbits per group. At *p* < 0.05 difference between the two groups was accepted.

## Results

### The mRNA expression of SPP1 in rabbits’ hepatic tissue

**Figure 3.**
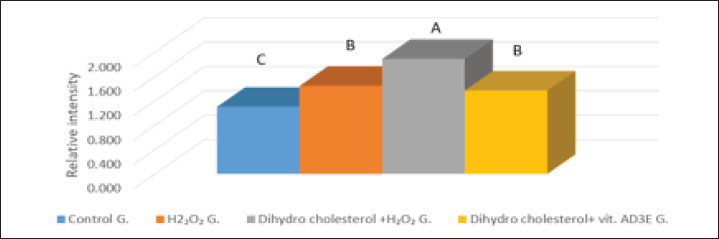
Effect of H_2_O_2_, dihydro cholesterol, and vitamin AD3E administration on SPP1 mRNA expression in the hepatic tissue using GAPDH as an internal control.

The PCR products for OPN (SPP1) amplification and GAPDH were (310 and 116) bp, respectively; the amplified levels of the mRNAs of SPP1 in hepatic tissues in the group that was fed an LD and exposed to H_2_O_2 _were increased but markedly decreased in the control group (Fig. 3). The mRNA OPN expression in the GIII was significantly increased when compared with other groups; the mean values ± SE of the GIII, GI, GII, and GIV, respectively, were (153.09 ± 4.009), (89.41 ± 0.58), (117.19 ± 9.11), and (111.08 ± 4.97). There is an insignificant increase in mRNA OPN expression in GII compared with GIV; the mean values ± SE of GII and GIV were (117.19 ± 9.11) and (111.08 ± 4.97). However, a significant increase in mRNA OPN expression was found in GII and GIV when compared with GI; the mean values ± SE of the GII, GIV, and GI were (117.19 ± 9.11), (111.08 ± 4.97), and (89.41 ± 0.58).

### Rate of gallstone formation in rabbits

GSs were not found in the gallbladder in any rabbits of the group’s GI and GII fed with a standard rabbit diet (Table 1). However, 100% and 50% of cholesterol GS were found in the gallbladders of rabbits in GIII and GV1, respectively, regardless of their sex, which considered the experimental groups fed with dihydrocholesterol for 3 weeks.

### Analysis of serum lipid profile

In the current study, the serum TC level was significantly increased in both GIII and GII when compared with G1; the mean values ± SE of serum TC levels of GIII, GII, and G1 were (112.8 ± 11.36), (99.60 ± 6.20), and (58.60 ± 2.65), respectively. While the serum TC level in G1V was insignificantly increased when compared with G1, the mean values ± SE of serum TC levels in G IV and G1 were (72.20 ± 7.996) and (58.60 ± 2.65), respectively (Table 2).

The serum TG level was significantly increased in groups GIII and GII when compared with G1; the mean values ± SE of serum TG levels of groups GIII, GII, and G1 were (127.6 ± 14.93), (104.6 ± 7.73), and (57.80 ± 9.906), respectively, while in G IV there was an insignificant increase in serum TG level when compared with G1. The mean values ± SE of serum TG levels of groups G IV and G1 were (77.80 ± 4.55) and (57.80 ± 9.906), respectively (Table 2).

The serum high-density lipoprotein-cholesterol (HDL-C) level was significantly lower in GIII compared with G1; the mean values ± SE of serum HDL-C in GIII and G1 were (26.0 ± 2.529) and (34.20 ± 3.99), respectively. There was an insignificant decrease in serum HDL-C in both groups GII and GIV when compared with G1; the mean values ± SE of serum HDL-C in GII, GIV, and G1 were (29.40 ± 1.20), (30.60 ± 0.509), and (34.20 ± 3.99), respectively (Table 2).

The serum low-density lipoprotein-cholesterol (LDL-C) level in all treated groups was significantly increased when compared to G1; the mean values ± SE of serum of groups GII, GIII, GIV, and G1 were (71.20 ± 16.67), (87.80 ± 9.759), (74.40 ± 9.479), and (18.20 ± 0.734), respectively (Table 2).

The serum VLDL-C levels in groups GII, GIII, and GIV were significantly higher when compared with G1; the mean values ± SE of serum VLDL-C levels in GII, GIII, GIV, and G1 were (26.80 ± 1.28), (29.80 ± 2.90), (20.40 ± 1.469), and (13.40 ± 1.43), respectively (Table 2).

The serum level of serum TC/HDL ratio was significantly increased in both G11 and GIII when compared with G1; the mean values ± SE of serum cholesterol/HDL levels of GII, GIII, and G1 were (3.38 ± .288), (4.33 ± .279), and (1.82 ± .338), respectively. While the serum level of TC/HDL ratio in G IV was insignificantly increased when compared with GI, the mean values ± SE of serum TC/HDL levels in G IV and GI were (2.416 ± 0.311) and (1.82 ± 0.338), respectively (Table 2). The serum level of LDL/HDL ratio in all treated groups GII, GIII, and G1V was significantly increased when compared with G1; the mean values ± SE of serum LDL/HDL ratio in GII, GIII, G1V, and GI were (2.60 ± 0.558), (3.558 ± 0.500), (2.172 ± 0.2406), and (0.572 ± 0.102), respectively (Table 2).

## Discussion

The cholesterol gallstone pathogenesis is considered multifactorial, and though OPN has been found in normal humans’ gallbladder bile and tissue, the role of OPN in forming GSs is still unknown [43]. In the current study, there is a relatively higher detection of mRNA OPN expression in hepatic tissue in the GIII when compared with the GII, GIV, and GI. Also, when comparing the GII, GIV, and GI, there is an increase in the mRNA OPN expression in their hepatic tissues. The results reported by Jin et al. [10] and Lin et al. [11] agreed with our data and suggested that hepatic mRNA OPN expression was significantly higher in the GSDs group than in GI. Whereas the results of the study by Yang et al. [44] disagreed with our data, which mentioned that protein and mRNA OPN expression was higher in normal tissue than in lithogenic gallbladder tissue.

**Table 1.** Rate of gallstone formation in rabbits.

Groups	Number of gallstone in rabbits	Number of gallstone rabbit	Rate of gallstone formation (%)
Control G.	6	0	0
H_2_O_2_G	6	0	0
Dihydrocholesterol G+ H_2_O_2_G	6	6	100
Dihydrocholesterol+ vitamin AD3E G.	6	3	50

**Table 2.** The mean ± SE of lipid profile in the four experimental groups.

Parameter mg/dl	GI	GII	GIII	G IV
S. TC	58.60 ± 2.6^5^b	99.60 ± 6.20^a^	112.8 ± 11.36^a^	72.20 ± 7.996^b^
S. TG	57.80 ± 9.906^c^	104.6 ± 7.7^3a,^b	127.6 ± 14.9^3^a	77.80 ± 4.55^c,b^
S. HDL-C	34.20 ± 3.99^a^	29.40 ± 1.20^a,b^	26.0 ± 2.52^9^b	30.60 ± 0.509^a,b^
S.LDL-C	18.20 ± 0.734^b^	71.20 ± 16.67^a^	87.80 ± 9.759^a^	74.40 ± 9.479^a^
S. VLDL-C	13.40 ± 1.4^3^c	26.80 ± 1.28^a^	29.80 ± 2.90^a^	20.40 ± 1.469^b^
TC/HDL ratio	1.82 ± 0.33^8^c	3.38 ± 0.288^b^	4.33 ± 0.27^9^a	2.416 ± 0.311^c^
LDL/HDL ratio	0.572 ± 0.102^c^	2.60 ± 0.558^a,b^	3.558 ± 0.500^a^	2.172 ± 0.2406^b^

*Different and same letters among groups in a row pointed to statistically significant and insignificant differences at *p* ≤ 0.05, respectively.

In this study, we added 1% dihydrocholesterol to the diet to induce cholesterol GSs. The rate of gallstone formation was 100% in the GIII when fed with 1% dihydrocholesterol for 21 days. While in the GIV, in addition to the administration of 1% dihydrocholesterol, we injected the rabbit with 0.1 ml of AD3E vitamins twice weekly to prevent or reduce the rate of GSs formation; therefore, the rate of GSs formation was 50% in the GIV. The results of Cui et al. [45] agree with ours, who mentioned in their study that the rate of GSs formation was 100%. In rabbits, when fed, a diet contains high cholesterol for more than 4 weeks. The prevalence of GSs formation in both males and females was similar in GIII, in which rabbits feeding on an LD (three of three females and three of three males) developed GSs. The results of Lin et al. [11] were consistent with our results; they mentioned in their study that the formation of GSs was the same in both genders; eighty percent of four out of five males and four out of five females of mice developed GSs when fed on an LD for 3 weeks when compared with the mice that fed on a normal diet.

In our results, we found a positive relationship between GS formation and serum lipid profile; GSs formation is proportional to hyperlipidemia, and the same finding was reported by Chen and Wu [46], and Atamer et al. [39] found a positive relation between GSDs and hyperlipidemia, which means that there were significantly high levels in the serum of TG, TC, and LDL-C in the GS group, while there were significantly low levels in the serum of HDL-C in the GS group when compared with the normal control group.

## Conclusion

It was concluded that when the rabbits were fed a high- cholesterol diet and exposed to H_2_O_2_, the incidence of gallstone formations reached a peak, and OPN mRNA expression was highly increased in their hepatic tissue, which proves that OPN is closely related to cholelithiasis and plays a significant role in the pathogenesis of GDs.

## List of abbreviations

cDNA, Complementary deoxyribonucleic acid; CGDs, cholesterol gallstone disease; DNA, Deoxyribonucleic acid; GAPDH, Glyceraldehyde-3-phosphate dehydrogenase; GDs, Gallstone disease; GIT, gastrointestinal tract; H_2_O_2_, Hydrogen peroxide; HDL-C, High-density lipoprotein-cholesterol; LD, Lithogenic diet; LDL-C, low-density lipoprotein-cholesterol; OPN, Osteopontin; RNA, Ribonucleic acid; ROS, Reactive oxygen species; RT-PCR, Reverse transcription-polymerized chain reaction; SPP1, Secreted phosphoprotein 1; TC, Total cholesterol; TG, Triglyceride.
